# ABCG2 Mediates Resistance to the Dual EGFR and PI3K Inhibitor MTX-211 in Cancer Cells

**DOI:** 10.3390/ijms25105160

**Published:** 2024-05-09

**Authors:** Chung-Pu Wu, Cheng-Yu Hung, Megumi Murakami, Yu-Shan Wu, Yi-Hsuan Chu, Yang-Hui Huang, Jau-Song Yu, Suresh V. Ambudkar

**Affiliations:** 1 Graduate Institute of Biomedical Sciences, College of Medicine, Chang Gung University, Taoyuan 33302, Taiwanyusong@mail.cgu.edu.tw (J.-S.Y.); 2 Department of Physiology and Pharmacology, College of Medicine, Chang Gung University, Taoyuan 33302, Taiwan; 3 Molecular Medicine Research Center, College of Medicine, Chang Gung University, Taoyuan 33302, Taiwan; aruhung@mail.cgu.edu.tw; 4 Department of Obstetrics and Gynecology, Taipei Chang Gung Memorial Hospital, Taipei 10507, Taiwan; 5 Laboratory of Cell Biology, Center for Cancer Research, National Cancer Institute, National Institutes of Health, Bethesda, MD 20892, USA; megumi.murakami@nih.gov (M.M.); ambudkar@mail.nih.gov (S.V.A.); 6 Department of Chemistry, Tunghai University, Taichung 40704, Taiwan; yushanwu@thu.edu.tw; 7 Department of Biochemistry and Molecular Biology, College of Medicine, Chang Gung University, Taoyuan 33302, Taiwan; 8 Liver Research Center, Linkou Chang Gung Memorial Hospital, Taoyuan 33302, Taiwan

**Keywords:** ABCG2, multidrug resistance, EGFR, PI3K, MTX-211

## Abstract

MTX-211 is a first-in-class dual inhibitor of epidermal growth factor receptor (EGFR) and phosphoinositide-3 kinase (PI3K) signaling pathways with a compelling pharmaceutical profile and could enhance the effectiveness of mitogen-activated protein kinase kinase (MEK) inhibitor therapy in colorectal tumors with KRAS mutations. However, the specific mechanisms contributing to the acquired resistance to MTX-211 in human cancers remain elusive. Here, we discovered that the overexpression of the ATP-binding cassette (ABC) drug transporter ABCG2, a prevalent mechanism associated with multidrug resistance (MDR), could diminish the effectiveness of MTX-211 in human cancer cells. We showed that the drug efflux activity of ABCG2 substantially decreased the intracellular accumulation of MTX-211 in cancer cells. As a result, the cytotoxicity and effectiveness of MTX-211 in suppressing the activation of the EGFR and PI3K pathways were significantly attenuated in cancer cells overexpressing ABCG2. Moreover, the enhancement of the MTX-211-stimulated ATPase activity of ABCG2 and the computational molecular docking analysis illustrating the binding of MTX-211 to the substrate-binding sites of ABCG2 offered a further indication for the interaction between MTX-211 and ABCG2. In summary, our findings indicate that MTX-211 acts as a substrate for ABCG2, underscoring the involvement of ABCG2 in the emergence of resistance to MTX-211. This finding carries clinical implications and merits further exploration.

## 1. Introduction

MTX-211 (also known as Mol 211, HY-107364), functioning as a dual inhibitor targeting both epidermal growth factor receptor (EGFR) and phosphoinositide-3 kinase (PI3K), exhibited potent in vivo growth-inhibitory effects against colorectal cancer models characterized by BRAF and KRAS mutations [1]. Furthermore, patients treated with a combination of MTX-211 and the mitogen-activated protein kinase kinase (MEK) inhibitor trametinib demonstrated a remarkable survival rate increase of over 400% compared to those treated with MTX-211 or trametinib alone [1]. Moreover, a separate study illustrated the synergistic therapeutic impact of the MTX-211 and trametinib combination on pancreatic cancer models. This combination therapy, as opposed to either agent administered alone, resulted in the heightened cell apoptosis and substantial inhibition of tumor growth in mice with mutations in KRAS and p53 [2]. A recent investigation revealed that MTX-211 hinders the synthesis of reduced glutathione (GSH) and displays anti-proliferative effects in bladder cancer [3]. Nevertheless, the mechanisms driving the acquired resistance to MTX-211 in human cancer cells remain unexplored. In addition to the well-documented resistance mechanisms against EGFR and PI3K pathway inhibitors, such as mutations [4,5] and the intricate crosstalk with other pivotal signaling pathways [6,7], drug efflux pumps might diminish the effectiveness of MTX-211 by reducing its intracellular concentrations in human cancer cells.

ABCG2, a member of the ATP-Binding Cassette (ABC) transporter superfamily, is renowned for its role in diminishing the chemosensitivity of cancer cells to conventional cytotoxic anticancer drugs. Through the utilization of energy sourced from ATP hydrolysis, ABCG2 actively expels a diverse array of therapeutic drugs from cancer cells, diverting them away from their intracellular targets [8,9]. Consequently, the higher levels of expression of ABCG2 frequently fosters the emergence of multidrug resistance (MDR) phenotypes in human cancer cells, leading to treatment inefficacy and relapse [8,10,11]. Furthermore, ABCG2 exhibits elevated expression in vital organs and critical blood–tissue barriers [10]. The increased expression of ABCG2 substantially impacts the pharmacokinetics of numerous therapeutic drugs, serving as an intrinsic defense mechanism against xenobiotics [10,12].

It is important to highlight that research has shown how the drug efflux activity of ABCG2 plays a pivotal role in restricting the bioavailability, distribution, and effectiveness of various targeted therapeutic drugs. These include, among others, inhibitors targeting the EGFR [13,14,15], PI3K [16,17], and mTOR pathways [18,19,20]. This study delved into the influence of ABCG2 on the emergence of the resistance or diminished susceptibility of cancer cells to MTX-211. Our findings revealed a notable decrease in the intracellular accumulation, efficacy, and cytotoxicity of MTX-211 in cancer cells with a high ABCG2 expression, a phenomenon that could be overcome by suppressing the activity of ABCG2. These results suggest that ABCG2 overexpression might play a role in MTX-211 resistance, emphasizing the potential necessity for combination therapy involving an ABCG2 modulator to enhance its clinical efficacy.

## 2. Results

### 2.1. Cells with High ABCG2 Expression Display Reduced Cytotoxicity to MTX-211

**Figure 1:**
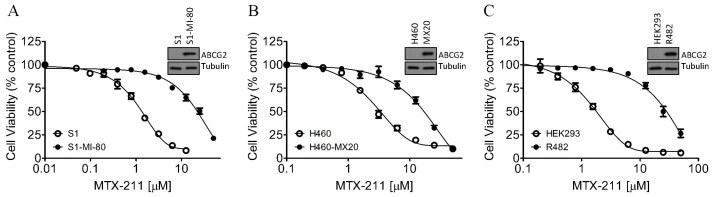
Distinct levels of toxicity induced by MTX-211 were observed between drug-sensitive and ABCG2-overexpressing multidrug-resistant cells. The cytotoxicity effects of MTX-211 were evaluated in three sets: (**A**) the drug-sensitive human colon cancer cell line S1 (open circles) and its ABCG2-overexpressing multidrug-resistant subline S1-MI-80 (filled circles); (**B**) the drug-sensitive human non-small cell lung cancer (NSCLC) cell line H460 (open circles) and its ABCG2-overexpressing multidrug-resistant subline H460-MX20 (filled circles); (**C**) parental HEK293 cells (open circles) and HEK293 cells transfected with human ABCG2 (R482-HEK293, filled circles) as described in Materials and Methods. Each data point represents the mean values obtained from more than three independent experiments, with error bars indicating the standard error of the mean (SEM). Insets are representative immunoblots showing ABCG2 and tubulin in parental and ABCG2-expressing cells.

To assess the influence of ABCG2 on the antiproliferative properties of MTX-211, we investigated its cytotoxicity across various pairs of cell lines comprising drug-sensitive strains and multidrug-resistant variants with ABCG2 overexpression. Our observations revealed more sensitivity to MTX-211 in parental human S1 colon cancer cells and H460 lung cancer cells compared to their respective ABCG2-overexpressing counterparts, S1-MI-80 (Figure 1A) and H460-MX20 (Figure 1B). Subsequently, we verified these findings by assessing the MTX-211 cytotoxicity in HEK293 cells and HEK293 cells transfected with human ABCG2 (R482-HEK293), demonstrating that ABCG2 confers resistance to MTX-211 in cancer cells (Figure 1A,B) and the ABCG2-transfected cells (Figure 1C). In Table 1, information on the IC_50_ and resistance factor (R.F.) values for MTX-211 across these cell lines are presented. The R.F. value quantifies the extent of cellular resistance to MTX-211 due to ABCG2, determined by dividing the IC_50_ value for MTX-211 in the drug-resistant variant by that in the respective parental line, ranging from approximately 5 to 21 as shown in Table 1. Notably, irrespective of tissue origin, MTX-211 exhibited significantly diminished cytotoxicity in cancer cells expressing ABCG2. Crucially, we found that attenuating the drug transport activity of ABCG2 fully restored the antiproliferative effect of MTX-211 in ABCG2-expressing cells. The ABCG2 reference inhibitor Ko143 resensitized S1-MI-80, H460-MX20, and R482-HEK293 cells to MTX-211 by approximately 10-fold, 7-fold, and 4-fold, respectively (Table 1). These findings indicate that the activity of ABCG2 plays a direct role in the development of resistance to MTX-211 in cancer cells.

### 2.2. Overexpression of ABCG2 Reduces the Intracellular Concentration of MTX-211 in Cancer Cells

**Figure 2:**
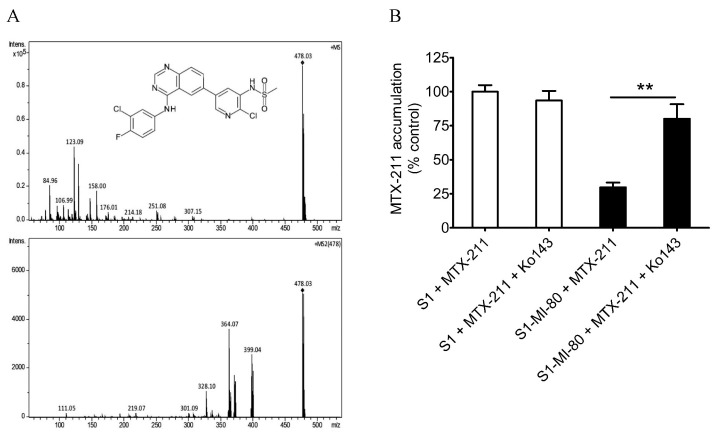
Intracellular accumulation of MTX-211 (with a molecular weight of 478 g/mol) in cancer cells overexpressing ABCG2. (**A**) The chemical structure (upper panel) and its fragment ions (lower panel) of MTX-211. (**B**) The quantification of intracellular MTX-211 concentration using LC-SRM/MS analysis in parental S1 cells (open bars) and ABCG2-overexpressing S1-MI-80 cells (filled bars) in the presence or absence of Ko143, as detailed in the Materials and Methods. The values represent the mean ± S.D. derived from a minimum of three independent experiments. ** *p* < 0.01, compared to treatment with Ko143.

One of the leading causes contributing to the reduced chemosensitivity associated with ABCG2 activity is the efflux of drugs thereby reducing their concentration within cancer cells. Accordingly, we investigated the intracellular levels of MTX-211 in parental cell line S1 and its ABCG2-overexpressing subline S1-MI-80, with or without the addition of 10 μM Ko143 using LC-MS/MS (Figure 2A), as detailed in Materials and Methods. As depicted in Figure 2B, the intracellular concentration of MTX-211 in S1-MI-80 cancer cells (5.92 ± 0.69 pmoles/μL) was notably lower than that in the parental S1 cancer cells (19.9 ± 0.95 pmoles/μL), a phenomenon that was susceptible to restoration by blocking the function of ABCG2 with Ko143.

### 2.3. Differential Inhibition of the EGFR and PI3K Signaling Pathways by MTX-211 in Drug-Sensitive and ABCG2-Overexpressing Cancer Cell Lines

**Figure 3:**
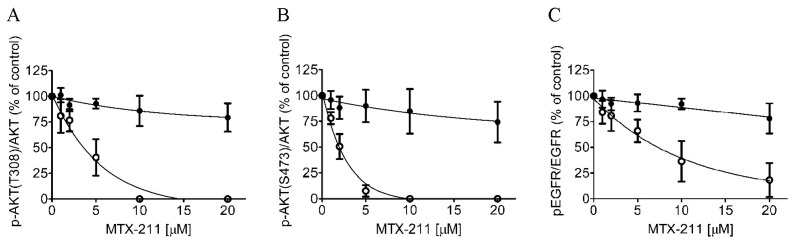
Differential effect of MTX-211 on PI3K/AKT/mTOR and EGFR signaling pathways in the S1 cell line and ABCG2-overexpressing S1-MI-80 subline. Quantification of relative phosphorylation levels of (**A**) AKT at T308 (pT308 AKT), (**B**) S473 (pS473 AKT), and (**C**) EGFR (pEGFR) in drug-sensitive parental S1 (open circles) and multidrug-resistant S1-MI-80 (closed circles) cancer cells treated with either DMSO (control) or MTX-211 (0–20 μM) for 2 h before being processed for immunoblotting. Human EGF (50 ng/mL) was introduced to the culture medium for 5 min to induce phosphorylation. The values represent the mean ± standard deviation, calculated from more than three independent experiments.

Since MTX-211 functions as a multi-targeting inhibitor intended to impede EGFR and PI3K signaling pathways [1], we examined its impact on EGFR and AKT phosphorylation within both parental cell line S1 and ABCG2-overexpressing subline S1-MI-80. As anticipated, MTX-211 effectively suppressed the phosphorylation levels of EGFR and AKT in both S1 and S1-MI-80 cancer cells in a dose-dependent fashion (Figure 3). Specifically, MTX-211 demonstrated the significant inhibition of AKT at both threonine 308 (pT308 AKT) and serine 473 (pS473 AKT), and EGFR phosphorylation in S1 cancer cells, exhibiting IC_50_ values of 2.72 ± 1.23, 1.50 ± 0.61, and 6.64 ± 1.27 μM, respectively (Figure 3, open circles). In contrast, its impact on ABCG2-overexpressing S1-MI-80 cancer cells revealed notably higher IC_50_ values exceeding 20 μM for the phosphorylation of AKT (T308), AKT (S473), and EGFR (Figure 3, filled circles). Of note, as reference inhibitors to achieve the complete inhibition of the PI3K signaling pathway and activation of EGFR, the PI3K/mTOR kinase inhibitor apitolisib (GDC-0980) [21] and EGFR inhibitor gefitinib at 10 μM were used, respectively.

### 2.4. Inhibition of the Efflux Activity of ABCG2 Restores MTX-211-Induced Apoptosis in Cancer Cells

**Figure 4:**
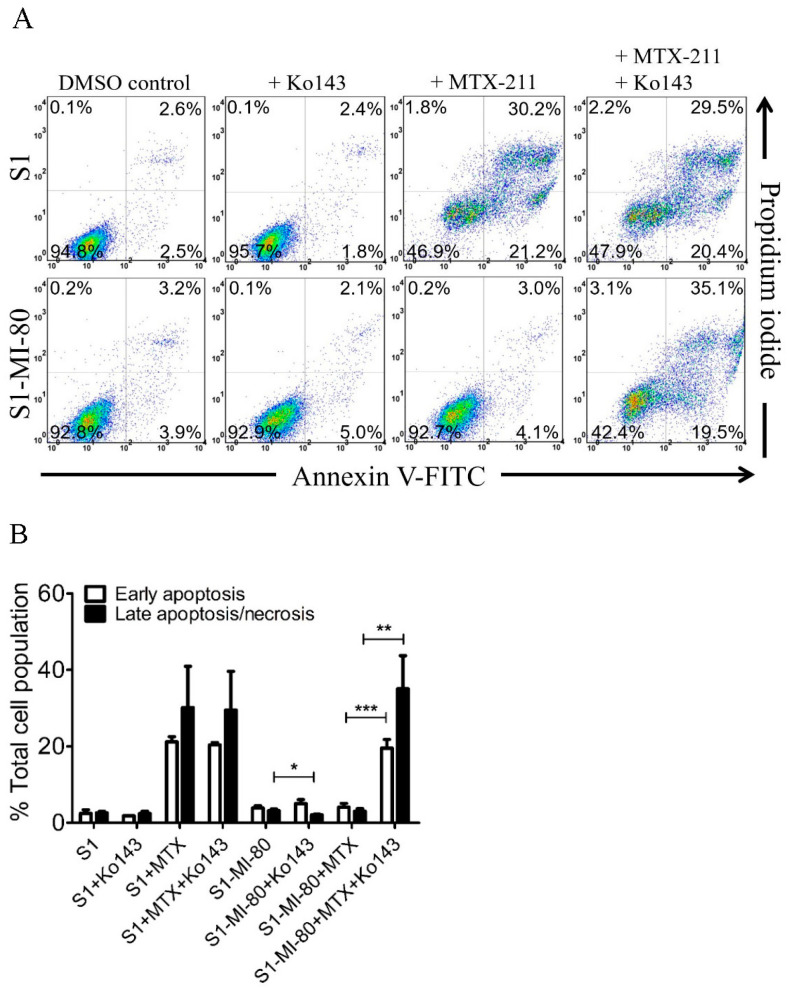
ABCG2 mitigates MTX-211-induced apoptosis in cancer cells. (**A**) The representative dot plots and (**B**) quantification results of the S1 cancer cell line and its ABCG2-overexpressing subline S1-MI-80 upon treatment with DMSO (DMSO control), Ko143 (+Ko143), MTX-211 (+MTX-211), or a combination of MTX-211 and Ko143 (+MTX-211 + Ko143). Cells underwent processing and analysis following the protocols detailed in Materials and Methods. Quantitative data are expressed as mean ± S.D. calculated from at least three independent experiments. * *p* < 0.05; ** *p* < 0.01; *** *p* < 0.001, compared to the same treatment in the presence of Ko143.

The induction of apoptosis by MTX-211 was examined in S1 and S1-MI-80 cancer cells. As anticipated, treatment with 20 μM of MTX-211 markedly elevated the overall apoptotic cell population from 5% to 51% in S1 cancer cells. Conversely, MTX-211 showed no significant impact on the total apoptotic cell count in S1-MI-80 cancer cells. Significantly, the ABCG2 inhibitor Ko143 (1 µM) notably increased the MTX-211-induced apoptotic cell count from 7% basal to 55% in S1-MI-80 cancer cells (Figure 4). The effect of MTX-211 on early and late apoptosis/or necrosis is shown in Figure 4B. These findings suggest that the ABCG2 inhibitor can restore MTX-211-induced apoptosis in cancer cells overexpressing ABCG2 by attenuating ABCG2 activity.

### 2.5. MTX-211 Stimulates the ATPase Activity of ABCG2

**Figure 5:**
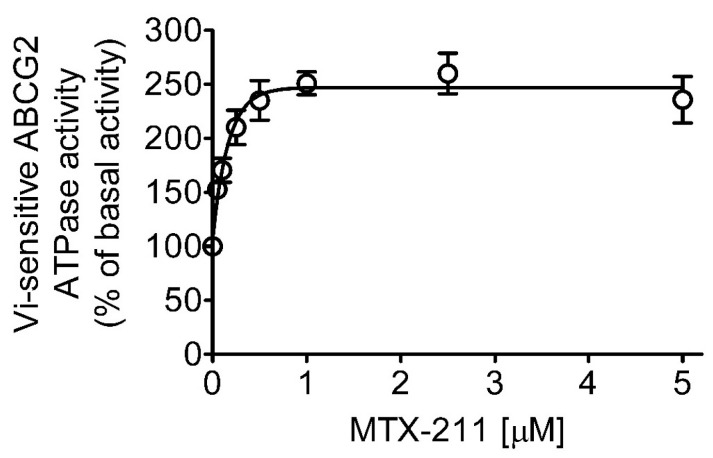
MTX-211 stimulates the ATP hydrolysis activity mediated by ABCG2. The impact of MTX-211 (0–5 µM) on the vanadate (V_i_)-sensitive ATPase activity of ABCG2 was evaluated through endpoint P_i_ release assay. This evaluation was conducted utilizing membrane vesicles obtained from ABCG2 baculovirus-infected High-Five insect cells, following the procedures outlined in Materials and Methods. The data are depicted as mean ± S.D. derived from three independent experiments.

Considering that ABCG2-mediated substrate transport relies on the hydrolysis of ATP and a majority of substrates stimulate the activity [10,11,17,22], we investigated whether MTX-211 affects the vanadate (V_i_)-sensitive ATPase activity of ABCG2 using membrane vesicles of High-Five insect cells expressing human ABCG2 protein, as detailed in Materials and Methods. Our findings revealed that MTX-211 augmented the ATPase activity of ABCG2 in a concentration-dependent manner (Figure 5), reaching a peak stimulation of 157% compared to the basal value of 116.58 ± 12.50 nmoles P_i_/min/mg protein (taken as 100%).

### 2.6. Docking of MTX-211 within the Drug-Binding Cavity of ABCG2 

To elucidate the interactions between MTX-211 and the substrate-binding domains within ABCG2, an in silico molecular docking analysis of MTX-211 using the inward-open structure of a homodimer of human ABCG2 (PDBID: 6VXJ) was conducted [23]. An examination of the lowest energy docking poses unveiled the anticipated sites of interaction between MTX-211 and various hydrophobic and aromatic residues situated within the transmembrane domains (TMDs) of ABCG2. The P_i_–P_i_ stacking interactions were predicted between the Phe^439^ phenyl moiety from opposing ABCG2 monomers and the quinazoline/pyridine moiety from MTX-211. Residues such as Val^442^ and Val^546^ may form hydrophobic and pi–alkyl interactions with chlorophenyl and quinazoline groups on MTX-211. 

## 3. Discussion

The novel EGFR and PI3K dual inhibitor MTX-211 demonstrated promising preclinical activity [2,3]. More importantly, Maust et al. highlight the potent growth-inhibitory properties of MTX-211 in BRAF-mutant and KRAS-mutant colorectal cancer models. Notably, patients administered the combination of MTX-211 and MEK inhibitor trametinib exhibited a survival rate considerably higher than those treated with either drug alone [1]. Nonetheless, the prospect of resistance emergence to MTX-211 poses a potential therapeutic obstacle in the future, with the mechanism behind such resistance remaining elusive. It is noteworthy that numerous independent research groups have documented the intricate interplay among EGFR, the PI3K signaling pathway, and ABCG2, highlighting how the efficacy of EGFR and PI3K inhibitors can be constrained by ABCG2 activity [15,17,24,25,26,27,28,29,30]. In the present study, our objective was to assess the influence of ABCG2 on the anti-proliferative efficacy of MTX-211. 

We observed that MTX-211 displayed inhibitory effects on cancer cell proliferation across various origins, with IC_50_ values falling within the range of approximately 1–8 μM, consistent with findings previously reported by Hu et al. [3]. However, we noted the resistance to MTX-211 in cancer cells overexpressing ABCG2 alongside HEK293 cells ectopically expressing human ABCG2 (R482-HEK293), compared to parental HEK293 cells (Table 1). Additionally, the impact of MTX-211 on PI3K signaling (Figure 3) and apoptosis (Figure 4) was notably diminished in ABCG2-overexpressing S1-MI-80 cancer cell lines compared to their parental counterparts. Nevertheless, the prolonged effect of MTX-211 treatment on ABCG2 expression in cancer patients remains to be elucidated. As the compromised sensitivity to chemotherapy associated with ABCG2 activity represents a pivotal factor contributing to the decreased accumulation of drugs within cancer cells, and that the ABCG2-mediated resistance to MTX-211 could pose a therapeutic challenge in the future, we evaluated whether inhibiting ABCG2 could overcome MTX-211 resistance in multidrug-resistant S1-MI-80 cancer cells. To explore this, we assessed the intracellular levels of MTX-211 (Figure 2A) in both S1 cells and its ABCG2-overexpressing counterpart, S1-MI-80, with or without Ko143, using LC-SRM/MS, as previously outlined [22]. As depicted in Figure 2B, the intracellular levels of MTX-211 in S1-MI-80 cancer cells were notably lower compared to those in the parental S1 cancer cells, and this disparity was effectively reversed by Ko143. Our results highlight that the reduced intracellular accumulation observed in S1-MI-80 cancer cells was substantially restored by Ko143, emphasizing the significant role of ABCG2 activity in the diminished effectiveness of MTX-211 in this cancer cell, as shown in Table 1. Both the cytotoxicity (Figure 1) and decreased intracellular concentration of MTX-211 in ABCG2 expressing cells (Figure 2) clearly demonstrates that this drug is a transport substrate of ABCG2.

**Figure 6:**
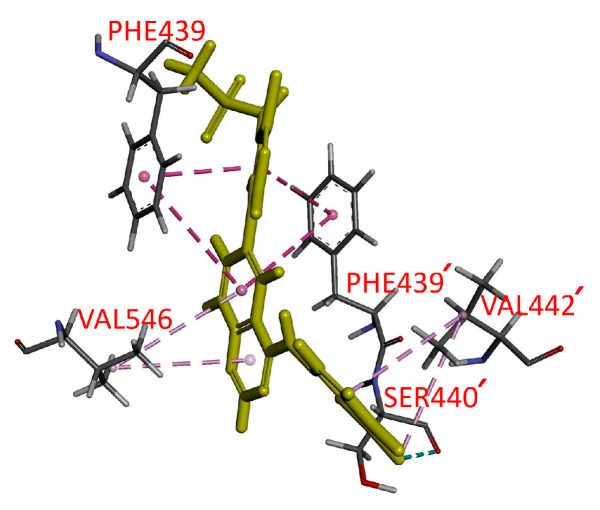
Docking of MTX-211 within the drug-binding pocket of ABCG2. Utilizing Accelrys Discovery Studio 4.0 software, the lowest energy binding mode of MTX-211 to the cryo-electron microscopy inward-open structure of human ABCG2 (PDB: 6VXJ) was predicted, following the procedures outlined in Materials and Methods. The molecular model of MTX-211 is depicted in stick representation (highlighted in yellow), while the atoms corresponding to interacting amino acid residues are color-coded as follows: carbon-gray, nitrogen-blue, oxygen-red, hydrogen-light gray, and fluorine-cyan. The proposed interactions are indicated by dotted lines. The residues from the monomer 2 of ABCG2 are indicated with prime.

Although an FDA-approved ABCG2 inhibitor is currently unavailable, our study demonstrates the potential principle that inhibiting ABCG2 function can restore the efficacy of MTX-211 in ABCG2-overexpressing multidrug-resistant cancer cells. Moreover, the observed stimulation of ATPase activity of ABCG2 by MTX-211 (Figure 5) supports the notion that MTX-211 acts as a substrate for ABCG2. Additionally, an in silico molecular docking analysis of MTX-211 with the inward-open structure of human ABCG2 (PDB: 6VXJ) (Figure 6) predicts the potential site of interaction between MTX-211 and the substrate-binding pocket of human ABCG2. In conclusion, our study underscores that MTX-211 functions as a substrate for ABCG2, suggesting that this transporter may contribute to the resistance against MTX-211 in cancer patients. Considering the profound impact of ABCG2 on the absorption and distribution of therapeutic agents [10,11], the ABCG2-mediated transport of MTX-211 could pose a substantial therapeutic obstacle for clinical use of this drug. Consequently, while clinically approved treatments to overcome ABCG2-mediated resistance are currently lacking due to issues such as toxicities [31] and the inadequate metabolic stability of inhibitors [32], further exploration is necessary to investigate potential drug combination strategies that might effectively overcome ABCG2-mediated resistance to MTX-211.

## 4. Materials and Methods

### 4.1. Reagents and Chemicals 

Cell culture media and supplements were sourced from Gibco/Thermo Fisher Scientific, Inc. (Waltham, MA, USA), including Roswell Park Memorial Institute medium 1640 (RPMI-1640), Iscove’s Modified Dulbecco’s medium (IMDM), Dulbecco’s Modified Eagle’s medium (DMEM), fetal calf serum (FCS), trypsin-EDTA, penicillin, and streptomycin. The Cell Counting Kit-8 (CCK-8) from Biotools Co., Ltd. (Taipei, Taiwan), and the Annexin V-FITC Apoptosis Detection Kit from BD Pharmingen (San Diego, CA, USA) were also employed. MTX-211 was provided by Selleckchem (Houston, TX, USA). Unless otherwise stated, all other chemicals used in the study were obtained from Sigma-Aldrich (St. Louis, MO, USA).

### 4.2. Cell Lines and Culture Conditions

The S1 human colon cancer cell line and H460 NSCLC cell line were cultured in RPMI-1640 medium. S1-MI-80, an ABCG2-overexpressing subline of S1, and H460-MX20, an ABCG2-overexpressing subline of H460, were cultured in RPMI-1640 medium supplemented with either 80 μM of mitoxantrone [33] or 20 nM of mitoxantrone [34], respectively. Human embryonic kidney (HEK293) cells transfected stably with either an empty pcDNA 3.1 vector (pcDNA3.1-HEK293) or human ABCG2 (referred to as R482-HEK293) were cultured in DMEM medium containing 2 mg/mL G418, as previously described [35]. All cell lines were maintained in a medium supplemented with 10% FCS, 2 mM _L_-glutamine, and Gibco penicillin-streptomycin at 37 °C in 5% CO_2_ humidified air. These cell lines were graciously provided by Dr. Susan Bates (NCI, NIH, Bethesda, MD, USA).

### 4.3. Cytotoxicity Assay

Cytotoxicity assessments followed established protocols [22]. Initially, cells were plated in 96-well flat-bottom plates at a density of 5000 cells per well in drug-free DMEM or RPMI-1640 devoid of drugs and incubated overnight at 37 °C in 5% CO_2_ humidified air. Following this, cells were exposed to MTX-211 either with or without Ko143 for 72 h. The subsequent analysis involved the utilization of MTT or CCK-8 reagents, as outlined in previous methodologies [22].

### 4.4. Ultra-Performance Liquid Chromatography (UPLC)-Selected Reaction Monitoring Mass Spectrometry (SRM/MS) Analysis of MTX-211 in Cancer Cells

The LC-MS/MS procedure for measuring the intracellular levels of MTX-211 was carried out and counted in accordance with a method outlined previously [22] with minor adjustments. Initially, 2 × 10^6^ cells were exposed to 10 μM of MTX-211 with or without 10 μM of Ko143 at 37 °C for 1 h. Following rinsing with cold PBS, cells were harvested and suspended in methanol at threefold volume, followed by overnight storage at −20 °C. Methanol extraction and subsequent centrifugation (10,000 rpm) at 4 °C for 30 min were performed on the cell lysates. The resulting supernatants underwent drying using a speed-vacuum-drying system and were reconstituted in 50% acetonitrile/H_2_O containing 0.1% formic acid for LC-MS analysis. The separation was accomplished using a Waters BEH C18 column (1.0 × 100 mm, 1.7 µm particles) with H_2_O (A, 0.1% formic acid) and acetonitrile (B, 0.1% formic acid) as the mobile phase, running at a rate of 0.06 mL/min. A gradient method was employed, as follows: initially, 10% B; at t = 0.5 min, 10% B; at t = 4.0 min, 35% B; at t = 6.0 min, 60% B; at t = 6.5 min, 90% B; at t = 8.2 min, 90% B; and at t = 8.5 min, 10% B, followed by a 3.5 min equilibration period. The column oven was maintained at 40 °C, while the autosampler was set at 12 °C. In positive mode, the quantitation using selected reaction monitoring (SRM) transition was performed at m/z 478.0→399.0 for MTX-211 using HCT ultra (Bruker Daltonik GmbH, Bremen, Germany). DataAnalysis 4.2 software was utilized for quantitative analysis based on peak areas. The MTX-211 standard, covering concentrations ranging from 62.5 fmol/µL to 4 pmol/µL, was prepared via serial dilution from the MTX-211 stock. Calibration curves, including an equal matrix background of cell lysates untreated with MTX-211, were established for LC-SRM/MS analysis. These calibration curves were employed to semi-quantitatively measure the intracellular accumulation of MTX-211 in cells after treatment with MTX-211, either with or without Ko143.

### 4.5. Immunoblotting

The cellular lysates underwent SDS-PAGE gel electrophoresis and subsequent transfer onto nitrocellulose membranes. The membranes underwent incubation with the following primary antibodies: anti-phospho-Akt (Thr308) (244F9) (#4056), anti-phospho-Akt (Ser473) (#9271), anti-Akt (Pan) (11E7) (#4685), anti-ABCG2 (#4477), anti-phospho-EGFR (Tyr1068) (#2234), anti-EGFR (D1D4J) (#54359), or anti-α-tubulin (#2144). Subsequently, they were treated with horseradish peroxidase-conjugated secondary antibody goat anti-rabbit immunoglobulin G (IgG). These antibodies were procured from Cell Signaling Technology, Inc., Danvers, MA, USA. Signal detection followed the procedure outlined previously [22].

### 4.6. Apoptosis Assays 

The conventional annexin V–FITC and propidium iodide (PI) staining technique was employed to assess cellular apoptosis [36]. In summary, cells underwent treatment with either DMSO (control), Ko143 (1 μM), MTX-211 (20 μM), or a combination of MTX-211 and Ko143 for a duration of 48 h. Subsequently, the cells were processed and analyzed following established procedures as previously outlined [22].

### 4.7. ATPase Assay

MTX-211 was evaluated across various concentrations (50–5000 nM) to test its effect on the ATPase activity of ABCG2 sensitive to vanadate was assessed using membrane vesicles derived from ABCG2-overexpressing High-Five cells. This evaluation employed the endpoint P_i_ release assay [37] and following previously established methodologies [22]. 

### 4.8. Docking of MTX-211 in the Substrate-Binding Pocket of ABCG2

The Accelrys Discovery Studio 4.0 software facilitated the generation of energy-minimized structure representing the inward-open conformation of the ABCG2 protein (PDB: 6VXJ) [23] and MTX-211 using the CHARMM force field at pH 7.4. MTX-211 was docked into ABCG2 using the CDOCKER module of the software. The respective interaction energy was computed and opted for the conformation exhibiting the lowest CDOCKER interaction energy, adhering to previously outlined methods [22].

### 4.9. Data Analysis 

Statistical data analysis and curve fitting were performed using KaleidaGraph version 4.5 from Synergy Software, Reading, PA, USA, and GraphPad Prism version 5 from GraphPad Software, La Jolla, CA, USA, respectively. Experimental values are expressed as mean ± standard deviation (SD) derived from a minimum of three independent experiments. A probability (*p*) value less than 0.05, as determined by a two-sided Student’s *t*-test, indicated statistical significance if there was a difference between mean values of experimental and control or the improvement in fit.

## Figures and Tables

**Table 1:** The effect of Ko143, a reference inhibitor of ABCG2, on the cytotoxicity of MTX-211 in ABCG2-overexpressing cells.

Cell Line	Transporter Overexpressed	IC_50_ ± SD [μM] ^1^ (R.F. ^2^)	
		MTX-211	MTX-211 + Ko143
S1	-	1.24 ± 0.09 (1)	1.38 ± 0.14 (1)
S1-MI-80	ABCG2	21.88 ± 2.32 *** (18)	2.11 ± 0.26 * (2)
H460	-	2.97 ± 0.32 (1)	1.82 ± 0.25 (1)
H460-MX20	ABCG2	14.94 ± 2.75 ** (5)	2.12 ± 0.19 (1)
pcDNA3.1-HEK293	-	1.36 ± 0.16 (1)	1.39 ± 0.22 (1)
R482-HEK293	ABCG2	27.94 ± 4.45 *** (21)	7.51 ± 0.90 *** (5)

Abbreviation: R.F.: resistance factor. ^1^ IC_50_ values are mean ± SD calculated from at least three independent experiments, in the presence or absence of 1 μM Ko143. ^2^ R.F. values were calculated by dividing the IC_50_ values of MTX-211 in multidrug-resistant cells by the IC_50_ values of MTX-211 in respective parental cells. * *p* < 0.05; ** *p* < 0.01; *** *p* < 0.001.

## Data Availability

The data presented in this study are available upon request from the corresponding author.
